# How to measure the effects and potential adverse events of palliative sedation? An integrative review

**DOI:** 10.1177/0269216320974264

**Published:** 2020-12-14

**Authors:** Alazne Belar, María Arantzamendi, Sheila Payne, Nancy Preston, Maaike Rijpstra, Jeroen Hasselaar, Lukas Radbruch, Michael Vanderelst, Julie Ling, Carlos Centeno

**Affiliations:** 1 Institute for Culture and Society-ATLANTES, Universidad de Navarra, Pamplona, Spain; 2 IdISNA, Pamplona, Spain; 3 Division of Health Research, Lancaster University, Lancaster, UK; 4 Department of Anaesthesiology, Pain, Palliative Medicine, Radboud University Medical Centre, Nijmegen, Netherlands; 5 Department of Palliative Medicine, Universitaetsklinikum Bonn, Bonn, Germany; 6 Department of Oncology, Laboratory of experimental radiotherapy, Katholieke Universiteit Leuven, Leuven, Flanders, Belgium; 7 European Association of Palliative Care, Vilvoorde, Belgium; 8 Clínica Universidad de Navarra, Departamento Medicina Paliativa, Pamplona, Spain

**Keywords:** palliative medicine, palliative care, terminal care, terminally ill, hospice care, patient comfort, empirical research, deep sedation, review, symptom assessment, sedation (as the MeSH Terms refers only to one type of sedation)

## Abstract

**Background:**

Palliative sedation is the monitored use of medications intended to relieve refractory suffering. The assessment of palliative sedation has been focused on the assess of the level of consciousness but a more comprehensive approach to assessment is needed.

**Aim:**

To understand how the potential effects and possible adverse events of palliative sedation in Palliative Care patients are measured.

**Design:**

Integrative review of most recent empirical research.

**Data sources:**

Cochrane Library, Embase, Medline, PubMed, and CINAHL were searched (2010–2020) using the terms sedation, palliative care, terminal care, assessment. Limits included studies in English and adults. Inclusion criteria were: scientific assessment papers, effects and complications of palliative sedation; patients with incurable illness.

**Results:**

Out of 588 titles, 26 fulfilled inclusion criteria. The Discomfort Scale-Dementia of Alzheimer Type and Patient Comfort Score were used to assess comfort. The Richmond Agitation-Sedation Scale and The Ramsay Sedation Scale are the most used to measure its effect. Refractory symptoms were assessed through multi-symptom or specific scales; except for psychological or existential distress. Delirium was assessed using the Memorial Delirium Assessment Scale and pain through the Critical Care Pain Observation Tool. The use of technical approaches to monitor effects is upcoming. There is lack of measurement of possible adverse events and variability in timing measurement.

**Conclusions:**

There are palliative care validated instruments to assess the sedation effect but this review shows the need for a more standardized approach when assessing it. Instruments should be used within an experienced and trained expert, providing a holistic assessment.

**What is already known about the topic?**

- In the context of patients with incurable disease palliative sedation is used for refractory symptom control.
- A minority of articles measure the effect of palliative sedation and current assessment of parameters of such effect is limited.
- The literature about palliative sedation measurement has mainly focused on medication use and level of sedation.

**What this article adds?**

- Discomfort Scale-dementia of Alzheimer Type (DS-DAT) and Patient Comfort Score (PCS) are assessment instruments being used to measure the effect of palliative sedation on patient comfort, the latter being validated for palliative care context.
- There is limited evidence on the timing of assessment, reported use ranges from daily assessment to six times per day, with often hourly measurements until adequate sedation is achieved.
- There is limited data available on the training and preparation of the health professional who has the responsibility for assessing refractory symptoms and palliative sedation.
- There is a lack of evidence, regarding measurement approaches or instruments for assessment of existential and psychological distress leading to palliative sedation; and also, for measuring adverse events.

**Implications for practice, theory or policy**

- Measurement instruments adapted to palliative care context should be used to assess palliative sedation, as these will facilitate practice improvement and comparability of the study’s results.
- Adding measurement instruments for comfort can contribute to assessing palliative sedation effects.
- A more standardized approach to assessing the effect of palliative sedation and possible adverse events, paying special attention to adequate training of health care professionals and timing of measurements, is needed to improve the quality of palliative sedation.

## Background

Patients nearing death can experience physical, psychological or existential discomfort that causes major distress. In some cases, symptoms can become refractory, which means that treatment options are exhausted either because they fail, the results are not available in sufficient time, or the risk-benefit ratio is no longer acceptable to the patient. In such cases, palliative sedation may be considered.^1–3^

The European Association of Palliative Care (EAPC) defined palliative sedation as “the monitored use of medications intended to induce a state of decreased or absent awareness (unconsciousness) in order to relieve the burden of otherwise intractable suffering in a manner that is ethically acceptable to the patient, family and healthcare providers” (p. 581).^3^ The EAPC highlights that different terms exist for palliative sedation. Most articles agree that palliative sedation needs to be administered exclusively to patients close to death who are suffering from refractory symptoms,^4–11^ with the aim of relieving suffering by administering medication.^1,3^ The administration of medication must be proportional to the relief of suffering,^10,12–18^ which means that the degree of sedation must not be deeper than necessary to relieve suffering.

Depending on the frequency of the administration of medication, palliative sedation can be intermittent or continuous. Palliative sedation can be light, intermediate or deep depending upon the levels needed to ensure comfort for the patient.^16^

In Europe, the proportion of deaths associated with palliative sedation is between 7% and 18%.^19,20^ However, the proportion of palliative sedation used is not easily assessed due to the existence of several definitions and the alternative terms used referring to it,^19,21^ the existing different types of sedation and the lack of standardized assessment instruments to measure it.^22^

Often in health contexts the gold standard used to measure distress and other symptoms is patient reported outcome measures (PROMs).^23^ In the case of light or intermittent palliative sedation, this is possible. However, it can be a challenge in cases of deep continuous sedation where there is impaired capacity to communicate.^2,24^ Therefore, there is a need for a comprehensive approach to explore options for assessment of palliative sedation; its effects and the possible subsequent adverse events.

In these cases, subjective assessments by professionals or via observer rating instruments in sedated patients are more commonly used.^2^ It is important that a selection of suitable methods is utilized to assess the effects of palliative sedation on patients and also the appearance of subsequent adverse events.

The last review by Brinkkemper et al. focused on observational scales to monitor symptom control and depth of sedation in patients requiring palliative sedation.^18^ They reported the scarce use of scales to measure the effect of palliative sedation, and they also suggested considering the frequency and timing of assessment. No other review has focused on the assessment of palliative sedation by considering a more inclusive approach where assessment goes beyond the use of observational instruments, for example including aspects such as adequacy, timing, and expertise.

The main aim of this article is to understand how the potential effects of palliative sedation in palliative care patients are assessed in the literature including a more inclusive methodology. The secondary aim is to explore the measurement of possible adverse events during palliative sedation.

## Design

An integrative review method was selected as the “broadest type of research review method allowing for the simultaneous inclusion of experimental and non-experimental research in order to more fully understand a phenomenon of concern” (p. 547).^25^ Whittemore and Knafl’s^25^ five stages were used to conduct the integrative review and the PRISMA framework^26^ was used to report findings.

**1. The problem identification stage.** There are reviews on observational instruments to monitor symptom control and depth of palliative sedation. Assessment is more than use of instruments so, how are the potential effects of palliative sedation and its possible adverse events assessed?
**2. The literature search stage** involved a systematic search strategy on five databases: Cochrane Library, Embase, Medline, PubMed and CINAHL. The last published systematic review on observational instruments in palliative sedation included articles from 1989 up to 2010,^18^ thus the parameters were from 1st January 2010 until 29th May 2020.

Three main concepts were combined: sedation, palliative care, and assessment.

Search strategies were revised with an expert librarian in biomedical databases and adjusted for each database. The key terms were sedation, palliative care, terminal care and assessment, as these were a robust and valid strategy to identify the relevant literature (Table 1). Established limits are shown in Table 1. A balance was made between the sensitivity and specificity of the search strategy (Supplemental Appendix 1). Experts who have been involved in research in the field suggested key articles on the topic and these were used to test the sensitivity of the search strategy.

**Table 1.** Search strategies on the different search engines.

Database	Search term
PubMed	#1 Sedation [all fields]
	#2 Palliative care [MeSH]
	#3 Terminal Care [MeSH]
	#4 Assessment [all fields]
	#5 #1 AND #2 OR #3 AND #4
Medline (WoS)	#1 Sedation [title]
	#2 “Palliative care” [topic]
	#3 “Terminal Care” [topic]
	#4 Assessment [title]
	#5 #1 AND #2 OR #3 AND #4
Embase	#1 Sedation [title]
	#2 “Palliative care” [abstract]
	#3 “Terminal Care” [abstract]
	#4 Assessment [abstract]
	#5 #1 AND #2 OR #3 AND #4
Cinahl	#1 Sedation [title]
	#2 “Palliative care” [abstract]
	#3 “Terminal Care” [abstract]
	#4 Assessment [abstract]
	#5 #1 AND #2 OR #3 AND #4
Cochrane Library	#1 Sedation [Title, abstract, key word]
	#2 “Palliative care” [Title, abstract, key word]
	#3 “Terminal Care” [Title, abstract, key word]
	#4 Assessment [Title, abstract, key word]
	#5 #1 AND #2 OR #3 AND #4
Limits:	
Published between 1st January 2010 and 29th May 2020.	
English language	
Participants over 18 years old	
Cochrane: limited specifically to Cochrane reviews as this database pulls data from Pubmed and Embase, and these databases had been already searched	
Medline: limit of “journal articles” (referring to empirical journal articles) or “reviews”	

Reference lists of included articles were explored to detect additional cited articles. Citations of the included articles were tracked to identify other eligible articles.^27^ In the case of review articles, as their search time was out of our time frame and included older articles, the reviews were used to identify articles.

The article eligibility stage was carried out according to inclusion and exclusion criteria (Table 2) using Covidence software, which allows blind reviewing of titles and abstracts by two independent reviewers. Then full text assessment was conducted. Discrepancies were managed through discussion with a third researcher. Articles were fully read to identify included articles. Reasons for exclusion of articles not meeting the inclusion criteria were systematically recorded.

**3. The data evaluation stage** entailed assessing the quality of articles using the Critical Appraisal Skills Programme (CASP) (2019) tool.^28^ A score was assigned to each of the 10 or 12 items assessed for qualitative articles or cohort articles, respectively (1: response is affirmative, 0: response is unknown or negative). The score was used only to provide an overview of the quality of the articles. The quality of protocol or case study articles were not assessed as there were no applicable checklists. Data extraction and quality assessment were conducted by two researchers. Each researcher was responsible for data extraction for half of the articles, and 10% of the articles was reviewed by a second blinded researcher.^29^ No substantial differences were found between researchers, and discussions helped clarify the inclusion criteria.

**Table 2.** Inclusion and exclusion criteria.

Inclusion	Exclusion
Scientific articles (experimental and non-experimental research) that give information about effects and complications considering also more subjective perspectives.	There is no specification on the instruments used to assess neither refractory symptom nor sedation.
Articles that focus on the assessment from the perspective of patients, health professionals and patients’ proxies.	Focus on ethical discussion about sedation.
Articles in all settings (e.g. home, hospital, palliative care institution).	Focus on health professionals’ knowledge/attitudes.
In those articles where participants are patients, they must be 18 years or over, with an advanced incurable illness (cancer and non-cancer) who required palliative sedation in order to control refractory symptom.	Children.
	Articles focusing on validating instruments that did not report patient data.
	Reviews based on articles published before 2010.

Both researchers used a predefined data extraction checklist that was pilot tested with five articles and adjusted (Supplemental Appendix 2). When multiple articles from the same study were identified these were presented following each other and separated by discontinuous dots (Table 3).

**Figure 1.**
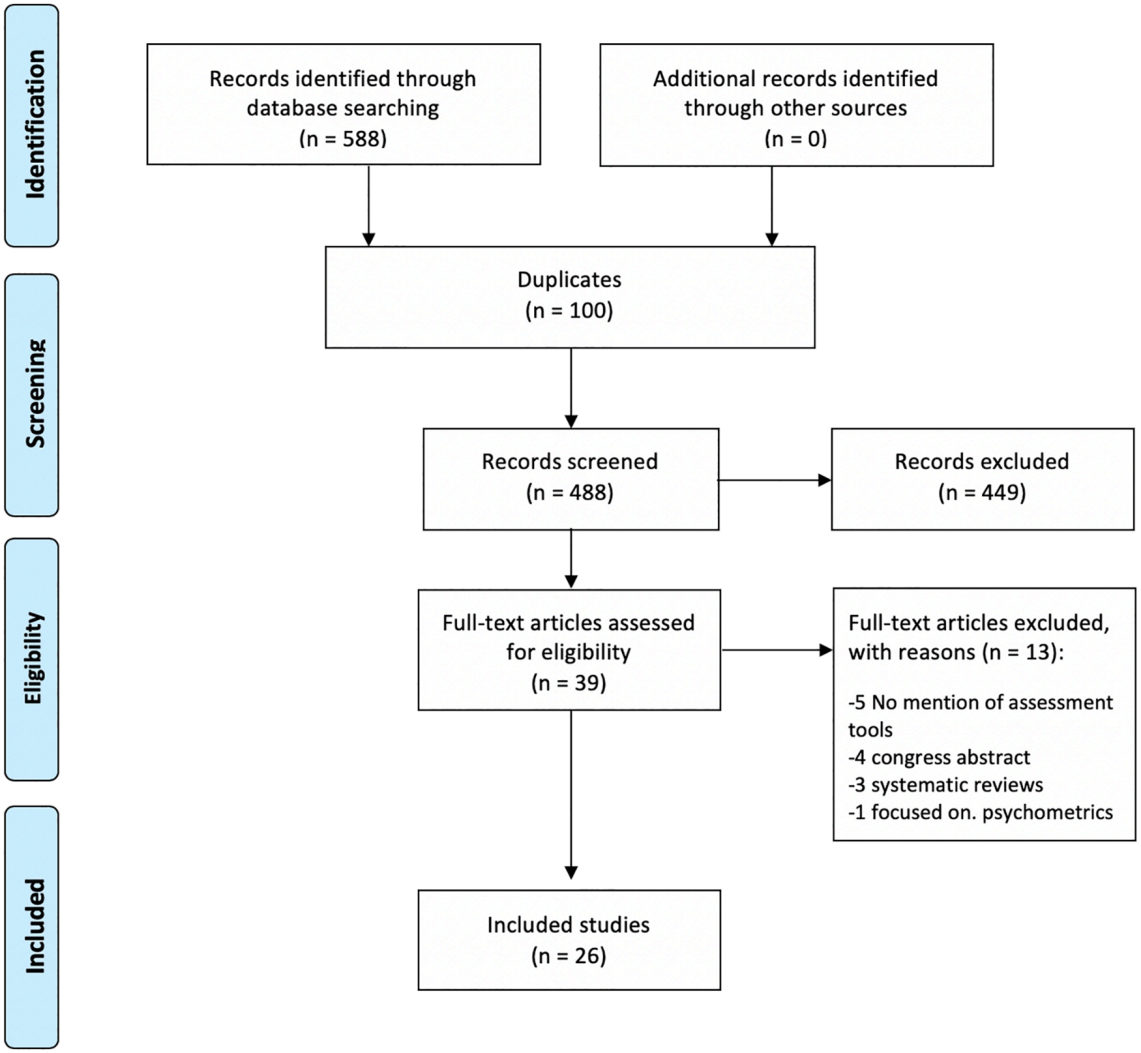
PRISMA flow chart from the search strategy.

**4. The data analysis stage** entailed data reduction and ordering the table with information from all the data extracted from the articles.^25^ Extracted data was compared and coded in two main areas: (1) study characteristics, quality assessment and type of sedation and (2) assessment process of palliative sedation and its indications, including also assessment responsibility and timing and adverse events. Initially, extracted data was compared; first quantitative studies and then qualitative studies, developing themes that were used to synthesize results. Two researchers developed these themes from the multiple primary sources.
**5. The presentation** of the review follows the PRISMA guidance including a flow diagram (Figure 1) and a structured presentation that comprehensively integrates evidence on: study characteristics, quality assessment and types of sedation mentioned, assessment of sedation and refractory symptoms and adverse events.

**Table 3.** Included articles characteristics.

Author, country, year	Objectives	Study design	Setting & context	Sample & general patients characteristics	Assessment	Casp score
Abdul-Razzak et al.^4^ (Canada)	To assess adherence of continuous palliative sedation practices with criteria set forth in local clinical guidelines, and to describe other features including prevalence, medication dosing, duration, multidisciplinary team involvement, and concurrent therapies.	Retrospective chart review (electronic and manual)	4 adult hospitals, 2 community hospices, 1 tertiary palliative care unit.	Patients who received a midazolam infusion at the end of life for deep CPST (*n* = 602). Gender: 50% men. Mean age: 65 years old	Richmond Agitation-Sedation Scale	6/12
					Riker Scale	
Alonso-Babarro et al.^30^ (Spain)	To investigate the frequency of PS use in cancer patients who had died at home, the reasons for PS use in these patients, the decision-making process for choosing PS, and the drugs administered to achieve PS.	Retrospective medical chart review	Madrid palliative home care team reviewed medical charts of all patients who received at-home care from the PHTC between January 2002 and December 2004.	Progressive incurable disease and high symptom burden, terminally ill cancer patients. Older than 18 years, at least once visited by the team and died at home. 370 cancer patient charts were reviewed and 245 patients died at home. 29 patients had PS. Age: mean age 58; Gender: 55% female	Ramsay scale	7/12
Arevalo et al.^31^ (The Netherlands)	To study the reliability and validity of observer-based sedation scales in palliative sedation.	Prospective evaluation	3 PC institutions in Amsterdam region of The Netherlands	All patients who received intermittent or continuous palliative sedation in a participating institution (December 2007–2010). 54 patients: 25 (46%) received intermittent sedation and 29 (54%) continuous sedation. Age: mean 73; Gender: 67% female.	Minnesota Sedation Assessment Tool	9/12
					Vancouver Interaction and Calmness Scale	
					Richmond Agitation-Sedation Scale	
					Sedation Score proposed in the Guideline for Palliative Sedation of the Royal Dutch Medical Association	
Azoulay et al.^12^ (Israel)	To describe the frequency and type of PS, the indications, associated medical diagnoses, the extent of patient and family involvement in the decision to initiate PS, and the improvement in symptoms following PS.	Retrospective, descriptive observational study	Hospice Unit of the Hadassah Hebrew University Medical Center (14 bed unit).	All advanced cancer patients derived to 1 hospice from different health services of the region in 2012 (*n* = 179 admitted, *n* = 38 received palliative sedation). Gender: 71% female, 29% male. Mean age: 73.3 ± 12.1 years old	Sedation assessed by PC professionals	4/12
					- Unconsciousness	
					- Proportionate	
					Intermittent	
Barbato et al.^32^ (Australia)	To determine the validity of the Richmond Agitation-Sedation Scale (RASS) and the Patient Comfort Score (PCS) in assessing sedation and comfort in unconscious patients.	Cohort study	1 palliative care unit at Port Kemble Hospital (15- bed unit).	Patients admitted for end-of-life care. Gender: 62% male. Age: range 41–97	Bispectral Index Monitor	7/12
					Richmond Agitation-Sedation Scale	
					Patient Comfort Score	
Benitez-Rosario et al.^6^ (Spain)	To assess the feasibility of a quality care project in palliative sedation.	Clinical audit. Quality-of-care methodology	Palliative care service (11-bed unit) within La Candelaria University Hospital.	Cancer patients 2007 (*n* = 60, 63.8%) and year 2008 (*n* = 63, 57.3%); Gender: men/women 2007 = 44/16; 2008 = 16/20; Age: range 2007 (36–90); 2008 (40–92)	Richmond Agitation-Sedation scale	5/12
					Palliative Prognostic Index	
					Patient Performance Scale	
					Karnofsky Performance Status	
					Oxygen saturation <90%	
					Death rattle	
					Severe pain	
					Existential suffering	
					Decision making: presence of advance care planning; patient and family information; expectation about sedation; verbal consent for PS; reasons for sedation; life expectancy at the moment of sedation and selected treatment	
Bruinsma et al.^33^ (Netherlands, Belgium, UK)	To explore relatives’ descriptions and experiences of continuous sedation in end-of-life care for cancer patients and to identify and explain differences between respondents from the Netherlands, Belgium, and the UK.	Qualitative	Patients who died in hospitals (oncology wards), palliative care units (PCU) (in Belgium), hospices (in the UK and the Netherlands), and in the community (at home)	Cancer patients who were administered palliative continuous deep sedation before dying. Patient characteristic: Male/female: 6/5 (BE); 7/6 (NL), 6/2 (UK); Age range: 30–92; all cancer; setting: community 4–5, Specialist PC or hospice 3–4, hospital 4–5 or 0. Bereaved relatives of patients with cancer who died after the use of continuous sedation until death (*n* = 32 of 84). Relative characteristics: male/female: 5/6 (BE), 4/9 (NL), 1/7 (UK). Partner: 5 (BE), 9 (NL), 4 (UK); Child: 3 (BE), 2 (NK), 4 (UK)	Relatives experience about PS	7/10
					Aide memoire: focused on relatives’ recollection of the care for the patient and of the use of sedation in particular	
Bush et al.^34^ (Canada)	To explore the validity and feasibility of a version of the RASS modified for palliative care populations.	Prospective study, using a mixed methods approach	36-bed inpatient in acute Palliative Care Unit (PCU).	10 consecutive patients with an agitated delirium or receiving continuous PS. Gender: 8 male and 2 females. Age: range 56-88 years. Disease: 9 metastasic cancer and 1 amyotrophic lateral sclerosis (ALS). Professionals: 5 palliative care physicians and eight nurses (seven BSNs and one PSN) participated.	Richmond Agitation-Sedation Scale-Palliative	7/12
					Nursing Delirium Screening Scale	
					Confusion Assessment Method	
					Delirium Rating Scale Revised-98	
					Memorial Delirium Assessment Scale	
Claessens et al.^7^ (Belgium)	To describe in detail the evolution of the level of consciousness of patients residing in palliative care units (PCUs) from admission until their day of death.	Prospective longitudinal and descriptive design.	Sample consisted of 3 PCUs in general hospitals, 1 hospice, 1 PCU in a university hospital, and 3 PCUs in general hospitals.	266 patients with incurable cancer with life expectancy <3 months, admitted from September 2004 to April 2005. 20 received palliative sedation. Gender: 54% male; Age: median age 72 years	- The Glasgow Coma Scale (GCS)	8/12
Gonçalves et al.^8^ (Portugal)	To study the practice of sedation by Portuguese palliative care teams.	Prospective multicentre study.	Portuguese PC teams. The four teams run: one inpatient care service, three hospital support care services, and one home care service; one of the teams run a hospital support care service and a home care service.	Of the 19 teams of Portuguese PC only 4 (21%) participated. 3-month period questionnaire from 5 April 2010 to 4 July 2010. 181 patients: 171 (94 %) were cancer patients and 10 noncancer patients. 27 (15 %) patients were sedated: 13 intermittently, 11 continuously, and 3 intermittently at first and continuously later. Sedated patients: male (19)/ female (8). Age median: 66.45 years (range 42–92)	- Consciousness Scale for Palliative Care	5/12
Hamatani et al.^35^ (Japan)	To survey the practice of palliative sedation in patients with HF at a tertiary cardiovascular referral center in Japan, and to investigate the feasibility of sedative agents in terminally ill heart failure patients	Retrospective study	Palliative Care Team at the National Cerebral and Cardiovascular Center	108 terminally ill HF patients who died during hospitalization. Gender: 78% male. Mean age: 70 years old	Vital signs: respiratory rate, oxygen saturation, blood pressure and heart rate.	8/12
					Richmond Agitation-Sedation Scale	
Imai et al.^13^ (Japan)	To investigate the effect of two types of palliative sedation defined using intervention protocols: proportional and deep sedation.	Retrospective cohort study of prospectively collected data	Consecutive terminally ill cancer patients who had received continuous infusion of midazolam according to intervention protocols for refractory symptoms in a PCU.	November 2015 to March 2017. 50 of total patients (12.6%) out of 398 admitted patients. Proportional sedation (*n* = 32): Age: 68.4 years; gender: Male (17)/female (15). Deep sedation (*n* = 18): Age: 70.2 years; Male (13)/female (5)	Richmond Agitation-Sedation Scale	10/12
					Support Team Assessment Schedule (STAS)^36^	
Maeda et al.^37^ (Japan)	To examine whether Continuous deep sedation (CDS) shortens patient survival and to explore the effect of artificial hydration during CDS on survival.	Secondary analysis of a large multicentre prospective cohort study	3 Sept 2012 to 30 April 2014, 58 PC institutions across Japan (19 hospital palliative care teams, 16 inpatient palliative care units, 23 home-based PC services.	1827 patient with a diagnosis of locally advanced or metastatic cancer were admitted and 269 (15%) received CDS before death. Included at admission and followed during 180 days or until death. Mean age 64.6. Gender: 61%	Confusion Assessment Method	9/12
					Modified Abbreviated Mental Test	
Maltoni et al.^16^ (Italy)	To investigate PS for refractory symptoms in different hospice case mixes in order to assess clinical decision-making, monitor the practice of PS, and examine the impact of PS on survival.	Observational prospective cohort study	Two Italian hospices	Patient admitted from October 2009 to June 2010 with palliative care needs. 72 (20%) patients underwent to palliative sedation. Median age: 68 years, 22% enrolled onto the underwent PS. Female: 51, 4%.	Richmond Agitation-Sedation Scale^38^	6/12
Mercadante et al.^39^ (Italy)	To assess the efficacy of a PS protocol, established by the HOCAI group in a preliminary investigator meeting. Secondary aim: analysis of the characteristics of these patients between 2 centers with different territorial facilities, any problems encountered by the teams, and level of satisfaction of the team and relatives.	Prospective study	Two home palliative care units	Advanced cancer patients who died at home (July–December 2012) to whom palliative sedation was administered. 219 patients were surveyed of whon 176 died at home. PS was performed in 24 of these patients (13.6%).	The Communication Capacity Scale	6/12
					Agitation	
					-The Agitation Distress Scale	
					Likert Scale (0–10)	
					- Pain	
					- Dyspnoea	
					- Agitated delirium	
					Psychological distress	
Monreal-Carrillo et al.^40^ (Mexico)	To characterize the level of consciousness in patients undergoing PS using Bispectral Index (BIS) monitoring.	Prospective observational study	Palliative care unit	27 hospitalized cancer with no further disease modifying treatment. Age: median 46 years Gender: 12 (60%) were female	- Ramsay Sedation Scale	9/12
					- Bispectral Index Score	
Park et al.^41^ (Korea)	To evaluate the association between CDS and survival in terminally ill cancer patients by analyzing matching data according to the relative probability of receiving CDS and using the beginning of CDS as the most appropriate time-point for assessing survival duration	Retrospective cohort study	Palliative care unit within Incheon regional cancer center.	974 oncological consecutive patients with terminal cancer whose expected survival was less than 3 to 6 months, according to the oncologist’s estimate, and who were referred to our palliative care unit from January 2012 to December 2016. Gender: 65% male. Mean age: 66.7 years old	- Eastern Cooperative Oncology Group Performance Status (ECOG-PS)	10/12
					-Palliative Prognostic Index	
					-Richmond Agitation-Sedation Scale	
Parra-Palacio et al.^35^ (Colombia)	To describe the sociodemographic and clinical characteristics of a group of cancer patients as well as prevalence, indications, time, and medications used for PS at a specialized PC unit at a cancer institution.	Descriptive prospective study	Clínica las Américas, hospitalized cancer patients.	66 patients included. Gender: 70% were women. Mean age: 61	- Ramsay Sedation Scale	6/12
					- Karnofsky Performance Scale	
					- Designed data collection instrument: demographic data, clinical information, Karnofsky index and symptoms and information on the implementation of PS, Ramsay, type of PS and start/ end time PS	
Pype et al.^17^ (Belgium)	To describe the occurrence and characteristics of suboptimal sedation in primary care and to explore the way general practitioners experience suboptimal palliative sedation in their practice.	Mixed methods study.	In Flanders, Belgium, 15 palliative home care teams (PHCT) are installed to advise and support GPs in caring for palliative patients at home. All 15 PHCTs were asked to participate. During a 6-month time period (May–October 2015), PHCTs were asked to register all cases of PS performed at home.	Seven out of 15 PHCTs agreed to participate. The 27 registered palliative sedations were administered to 23 men and 4 women, 21 of them were oncological patients, mean age was 71 year (range 47–91). Out of the 11 GPs who performed one of the identified cases of suboptimal sedation, 7 agreed to be interviewed, mean age was 44 years, 4 were female.	- Richmond Agitation-Sedation scale	7/12
Stiel et al.^42^ (Germany)	To collect contents from provided documents used in clinical practice of PS in Germany, to describe common core elements and particularities of these in service documents, and to compare, on the one hand, to what extent the documents match the recommendations from the EAPC framework and, on the other hand, how these documents used in clinical practice expand contents from the EAPC framework.	Survey	In September and October 2012, 605 contact persons from PCU 261), H (197), SAPV (127), and SAPPV (20) listed in national address registers were invited via e-mail to participate, each with one representative, in a national survey on the clinical practice of PS.	An overall response rate of 37.2% was achieved. 4 questionnaires excluded, resulting in *n* = 221 data sets. In total, 58 documents uploaded. After re-allocation of all documents to 4 respective document sets, duplication, exclusion of non-specific contributions for PS, the following distribution was used for further analysis. Ultimately, 307 content units from 52 documents were coded.	- Richmond Agitation-Sedation Scale	7/12
					- Ramsay Sedation Scale	
					- Beobachtungsinstrument für das Schmerzassessment bei alten Menschen mit Demenz BISAD	
					- The Critical Care Pain Observation Tool	
					- Edmonton Symptoms Assessment Scale	
					- Symptom and problem checklist of the Minimal Documentation System	
Six et al.^24^ (Belgium)	The aims are: (a) to better understand how unconscious palliative sedated patients experience the last days of life; (b) to firm out if they are really free of pain; (c) to evaluate to what degree assessments of comfort based on behavioral observation are in line with the results from a brain function monitor and ANImonitor; (d) to find out if changes in the measured depth of sedation can be experienced by patients, caregivers and relatives.	Study protocol	Modal hospitals and modal homes for the elderly	40 patients in last week of life.	- Awareness 3 VAS: no aware-completely aware	Not mentioned
					- Communication 3 VAS: no communication possible-full communication possible	
					- Comfort: 3 VAS no pain-severe pain	
					- Heart rate variability using an ANI monitor and electroencephalography	
					- Behavioral Pain Scale Non-Intubated	
					- Critical Care Pain Observational Tool	
					- Neurosense	
					- Richmond Agitation-Sedation Scale	
					- Modified Edmonton Symptom Assessment Scale	
Six et al.^43^ (Belgium)	Case presentation	Case	Palliative ward of a general hospital in Flanders, Belgium,	A woman in her mid-80s admitted (in 2017) to the palliative ward of a general hospital in Flanders.	- Heart rate variability using an ANI monitor	Not mentioned
					-Neurosense	
					-Ramsay Sedation Scale	
Song et al.^9^ (Korea)	_	Case presentation	Inpatient hospice unit.	Two cases of refractory severe cancer pain with psychological anguish controlled successfully by intermittent IPS for the long time. Gender: 50% female; Age: 46- and 65 years old.	- Pain with numeric Ratings Scale	Not mentioned
					- Pain that later was followed by FACES Pain Rating Scale	
Van Deijck et al.^10^ (The Netherlands)	To identify patient-related determinants of the administration of CPS at admission to a hospice or nursing home-based PC unit.	Prospective observational multicentre study	Six hospices and three nursing home PCUs in The Netherlands, follow up period of 3 months.	803 patients, life expectancy < 3 months, admitted to the participating hospices or PCUs, 503 patients gave written informed consent. 106 were sedated. Age: half of the population consisted of patients aged 76 years and older; Gender: 52% were female.	- Glasgow Coma Scale	11/12
					- Karnofsky Performance Scale	
					- Edmonton Symptom Assessment Scale	
Van Deijck et al.^2^ (The Netherlands)	To identify the course of discomfort using the DS-DAT in patients receiving CPS, admitted to a hospice or nursing home-based PC unit. A secondary goal was to identify patient-related determinants of discomfort in last hours of life of sedated patients.	Prospective observational multicenter study between march 2011 and December 2012	Six hospices and three nursing home PCUs in the Netherlands, with a follow up period of 3 months.	106 sedated patients, life expectancy < 3 months. Gender: 53% female. Age: 90% were >55 years old.	- Discomfort scale-dementia of Alzheimer type	11/12
					- Diagnostic and Statistical Manual of Mental Disorders, fourth edition	
Won et al.^44^ (Korea)	To identify the clinical patterns of palliative sedation according to a prescribed protocol and assessment tools.	Observational study	Hospice center affiliated with a tertiary medical center in Korea.	306 consecutive cancer patients at a hospice ward of a hospital. Gender: 68% male. Mean age: 65.8 years old	-Vital signs: blood pressure, respiratory rate, and oxygen saturation	7/12
					-Richmond Agitation-Sedation Scale	
					-Quality of sleep: ad hoc symptom-based grading scale ranging from grade 1 to grade 5	

## Results

A total of 588 articles were screened, 26 met the inclusion criteria. Two articles were part of the same study although they were referenced independently.^2,10^ Citation tracking of the 26 included articles was conducted in PubMed with no additional inclusions.

### Included study characteristics, quality assessment and type of sedation

The 26 articles originated from 14 countries including Belgium and Netherlands (*n* = 4); Korea and Japan (*n* = 3); Canada, Italy and Spain (*n* = 2); Australia, Columbia, Germany, Israel, Mexico, Portugal, and the United Kingdom (*n* = 1). The main characteristics of the articles are presented in Table 3.

Fourteen articles reflected work conducted in a single site.^6,9,11,13,24,30,32,34,35,40,43^ Ten studies included multiple sites within the same country or region^2,7,8,10,16,17,31,37,39,42^ and one included an international collaboration between three countries (Belgium, United Kingdom, and the Netherlands).^15^ The majority of studies were conducted in palliative care services. Eight articles included sedated patients with non-malignant disease,^2,8,10,17,31,32,34,45^ three did not specify a diagnosis^4,24,42^ and the rest included only cancer patients.

Fourteen articles reported on cohort studies.^2,6,7,10,13,16,31,32,35,37,40,41,44,45^ Seven studies used a cross-sectional design.^4,8,12,30,39,42,44^ There were two mixed method studies^17,34^ and two case studies^9,43^ and one qualitative methods study.^33^ Fifteen articles reported collecting data prospectively with samples ranging from 10 to 269 participants.^2,7,8,10,13,15–17,31,32,34,35,37,39,40^ Six studies were conducted retrospectively through chart or clinical documents review.^4,12,13,30,41,45^

The quality of articles was relatively good^7,13,16,17,30–35,37,39,40–42,44,45^ except for three studies with lower scores (scoring lower than 6/12).^6,8,12^ The two articles of van Deijck et al. stand out for their excellence (CASP 11/12).^2,10^

Context and palliative sedation types need to be considered as background information when considering its assessment. All the articles clearly set the use of sedation in the context of end of life, but there are nuances regarding timing. This varied between the last 6 months of life of an incurable disease^12,41^ to the last phase of terminal illness,^2,7,13,37,43^ which could be specified as a life expectancy of 2 weeks or less^10,15^ or last days or hours of life.^44^

Five articles mentioned cases of intermittent sedation,^8,9,12,35,44^ the others referred to continuous sedation, although using a variety of terms: palliative sedation,^6,7,16,17,24,30–32,34,39,40,42,43,45^ palliative sedation therapy,^6,13,37^ continuous palliative sedation therapy,^4^ continuous sedation^8,33,35^ and proportional palliative sedation.^12^ The concept of proportionality is mentioned in several articles^10,12,13,16,17,33^ relating the level of sedation to the degree of patient symptom control needed. Some articles mention specific outcome measures such as: describing suboptimal continuous deep sedation considering their own definition^17^; measuring the quality of sleep using an *ad hoc* symptom-based grading scale or palliative sedation success considering their set standards^44^ or the quality of the administered sedation regarding the patient comfort and physician estimation of success.^2^

### Assessment process of palliative sedation and its indications

This process refers to the instruments used, the people involved in the assessment, the timing of assessments and related-adverse events of palliative sedation (Table 3). Summary information about the instruments mentioned below is presented in Table 4.

**Table 4.** Description and validation information about identified instruments.

Scale*	Short description	Validated for PC	Number of included articles using
Refractory symptoms			
Behavioral Pain Scale non intubated^46^	What does assess with 9 four-point items. Summed scores range from 0 (observed discomfort)- 27 (high level of discomfort)	No, validated with ICU patients	1
Confusion Assessment Method^47^	Assesses delirium considering 9 aspects through 12 items	No, validated in ICU patients	2
Critical Care Pain Observational Tool^48^	Assesses pain and distress in patients with a lowered consciousness through 4 items each evaluated on a 0-2 scale	Yes	2
Delirium Rating Scale-Revised 98^49^	Assesses delirium through 13 items scoring between 0-3 each	No, validated in patients with delirium, dementia, schizophrenia, depression, psychiatric illness	1
Diagnostic and Statistical Manual of Mental Disorders^50^	Manual about a mental health disorder	No, validated in patients with psychiatric illness	1
Edmonton Symptom Assessment Scale^51^	10 item symptom (pain, fatigue, nausea, depression, anxiety, drowsiness, appetite, wellbeing, shortness of breath, others) scale scoring from 0 to 10 each	Yes	2
Eastern Cooperative Oncology Group Performance status^52^	Measures performance status ranging from 0 (fully active) to 5 (death)	No, validated in cancer patients	1
Faces Pain Rating Scale^53^	Assesses pain with a range from happy face at 0 (no hurt) to a crying face at 10 (worst pain. Imaginable)	No, validated in pediatrics	1
Karnofsky Performance Status Scale^54^	Measures performance status through 11 levels ranging from 0 (death) to 100 (active)	Yes	3
Memorial Delirium Assessment Scale^55^	Measures delirium through 5 individual items with a 0–3 score each	Yes	1
Minimal Documentation System^56^	10 item symptom (pain, nausea, vomiting, dyspnea, constipation, weakness, appetite loss, tiredness, feeling depressed, anxiety, others), scale scoring on 0 (none) to 3 (severe) score, and question on well-being scoring of −2 (very bad) to 2 (very well)	Yes	1
Modified Abbreviated Mental Test^57^	Assesses cognitive impairment considering 10 items with a maximum of 1 point each. A score 7–8 or less suggest cognitive impairment	No, validated in elderly patients with dementia	1
Modified Edmonton Symptom Assessment Scale^58^	10 item symptom scale scoring from 0–10 each	Yes	1
Palliative Performance Scale^59^	Considers the performance scale in 11 levels from 0% (death) to 100%(active) in 10 percent increments considering five functional dimensions	Yes	1
Palliative Prognostic Index^60^	Assesses prognostic with the sum of Palliative Performance Scale with other four clinical variables	Yes	2
Support Team Assessment Schedule^36^	Assesses overall symptom control: 0 (None) – 4 (severe and continuous overwhelming symptom(s)	Yes	1
Palliative sedation			
*Sedation*			
Agitation Distress Scale^61^	Considers agitation with 6 items: frequency, extent, content of physical restlessness, psychological instability, hallucinations and delusions and sleep disturbance	Yes	1
Guideline for Palliative Sedation by the Royal Dutch Medical Association^62^	Assess sedation divided into 3 levels from “basic brain function affected” to “eyes closed, arousable only by physical stimuli”	Yes	1
Minnesota Sedation Assessment Tool^63^	Assesses sedation through 3 subscales evaluating them with a 1–6 score	No, validated in ICU patients	1
Ramsay Sedation Scale^64^	Scores sedation from 1 (agitated and restless) to 6 (no response to light, glabella tap or loud noise)	No, validated in ICU patients	5
Richmond Agitation-Sedation Scale^38^	Scores sedation and agitation from +4 (combative) to −5 (unarousable)	No, validated in ICU patients	12
Richmond Agitation-Sedation Scale-Palliative^34^	Scores sedation and agitation from +4 (combative) to −5 (unarousable)	Yes	1
Riker Sedation Agitation Scale^65^	Scores sedation and agitation from 7 (dangerous agitation) to 1 (unarousable)	No, validated in ICU patients	1
*Consciousness*			
Communication Capacity Scale Score^61^	Evaluates 5 main items: conscious level, ability to answer open- and close- ended questions, achievement of voluntary communication and movement. Its overall score ranges from 0 to 17	Yes	1
Consciousness Scale for Palliative Care^66^	Evaluates consciousness from 1 (awake) to 6 (does not react)	Yes	1
Glasgow Coma Scale^67^	Evaluates coma status based on 3 separate subscales, ranging from 3 (deep comatose) to 15 (normal consciousness)	No, validated in ICU patients	2
Vancouver Interaction and Calmness Scale^68^	Assesses sedation through 2 subscales: interaction and calmness including different items evaluating on 1 (strongly disagree) to 6 (strongly agree)	No, validated in mechanically ventilated adults	1
*Comfort*			
Discomfort Scale-dementia of Alzheimer Type^69^	Evaluates discomfort with 9 four-point items and summed scores range from 0 (no observed discomfort) to 27 (high level of observed discomfort)	No, validated in dementia of Alzheimer Type	1
Patient Comfort Score^70^	Evaluates comfort ranging from 0 to 10: 0 = complete comfort; 1–4 = mild discomfort; 5–6= moderate discomfort and 7–10= severe discomfort.	Yes	1

* The reference numbers included in this table belong to the original scale reference.

#### Indications for palliative sedation

Several refractory symptoms such as pain, delirium, dyspnoea or vomiting as well as psychological and existential distress were registered as main reasons for starting palliative sedation. Some articles reported general assessment of symptoms by instruments validated in palliative care, containing multiple symptoms such as anxiety, depression, nausea or shortness of breath. General symptom assessment instruments such as the Support Team Assessment Schedule (STAS),^13^ the ten-item Edmonton Symptom Assessment System (ESAS),^42^ the Modified Edmonton Symptom Assessment Scale (M-ESAS)^24^ and the Minimal Documentation System (MiDOS) were used.^42^ Some other articles used team developed numerical instruments to measure pain,^9,24,39,42^ nausea,^42^ vomiting,^42^ dyspnoea,^39,42^ anxiety,^42^ psychological aspects,^39,42^ disorientation,^42^ and agitated delirium^39^ in sedated patients.

During sedation, delirium and pain were the most frequently assessed symptoms using validated instruments.^9,24,34,36,37^ Concerning the assessment of delirium, the articles reported the use of various instruments. The Memorial Delirium Assessment Scale (MDAS) is the only scale validated in palliative care patients.^34^ This instrument quantifies the severity of delirium over an extended time frame by rating ten individual items at a 4-point scale. Other articles used the Confusion Assessment Method (CAM)^34,37^ and the Delirium Rating Scale-Revised-98.^34^ These are validated instruments, in non-palliative care patients, which explore delirium by scoring aspects such as consciousness, attention/concentration, orientation, behavior and psychomotor activity (Table 4).

According to the Diagnostic and Statistical Manual of Mental Disorders delirium was determined in the article of van Deijck et al.^2^ Finally, the Modified Abbreviated Mental Test^37^ validated to assess dementia in older patients, was also used for assessing patients mental status, by considering the orientation and consciousness.

Pain was measured as an item within general symptom assessment instruments, such as ESAS and its modified version (M-ESAS), but also with specific pain assessment instruments. Specific instruments validated to be used for pain measurement were: (a) The Faces Pain Rating Scale with a series of faces representing no pain until the worst pain imaginable^9^; (b) the Critical Care Pain Observational Tool which considers the facial expression, body movements, muscle tension and the compliance with the ventilator or vocalisation^24,42^ and (c) in the Behavioral Pain scale for non-intubated patients facial expression, movements of upper limbs and compliance with ventilation or the vocalization were scored.^24^ From all reported instruments, the Critical Care Pain Observational Tool,^24,42^ the ESAS^10,42^ and the M-ESAS^24^ are validated for palliative care.

Assessment of life expectancy was performed using various instruments including: the Palliative Prognostic Index 10 (PPI)^6,41^; Palliative Performance Score (PPS)^6^; functional status with Karnofsky Performance Status Scale (KPS)^6,10,35^; Eastern Cooperative Oncology Group performance status scale; and an observer-rated scale of physical ability.^41^

Some studies reported assessment of vital signs when presenting their results on palliative sedation.^6,44,45^

#### Palliative sedation assessment

Most articles explained how they monitored sedation and in which domain: level of sedation, comfort level or symptom control.^2,6–8,10,13,16,17,24,30–34,37,39,40,42,43^ The majority also reported the use of the Richmond Agitation-Sedation Scale^6,13,16,17,24,31,32,41,42,44,45^ or the Ramsay Sedation Scale to objectify the effects of palliative sedation.^30,35,40,42,43^

The Richmond Agitation-Sedation Scale (RASS) is an observational scale that assesses the level of sedation and agitation without requiring patient input. It was validated in intensive care unit patients^38^ and adapted for a population with palliative care needs, calling it Richmond Agitation Sedation Scale-Palliative (RASS-PAL).^34^ Both instruments measure the patients’ level of sedation and agitation, scoring from +4 (combative) to −5 (not arousable).

Although not validated in a palliative care population, the Ramsay Sedation Scale is often used to measure effects of palliative sedation^64^ by scoring the patients’ sedation level within six categories ranging from severely agitated to not responsive.

Less frequently mentioned instruments for assessment of sedation levels, but validated in palliative care, are the assessment presented in the Guideline for Palliative Sedation by the Royal Dutch Medical Association,^31^ which considers sedation and the response to stimuli; the Consciousness Scale for Palliative Care,^8^ which assesses the consciousness level through stimulation, and the Agitation Distress Scale assessing agitation through observation of the patient.^39^

Specific instruments validated in other populations also utilized in sedation monitoring are the Minnesota Sedation Assessment Tool (MSAT)^31^ which scores the motor activity and the arousal of patients, and the Riker Sedation Agitation scale that considers the agitation and sedation of the patient.^39^

Effects of sedation have also been measured by considering the consciousness of the patient with the Glasgow Coma Scale recording eye opening and the motor and verbal response^7,10^; the Vancouver Interaction and Calmness Scale (VICS) considering the interaction with the environment^31^; and the Communication Capacity Scale Score assessing interaction level of the patient.^39^

Few clinical articles use comfort as an outcome measurement in sedated patients. Comfort of the sedated patients was measured through the Discomfort Scale-Dementia of Alzheimer Type (DS-DAT),^2^ based on observing different behavioral indicators; and the Patient Comfort Score (PCS),^70^ that considers pain and level of consciousness.

In some articles,^24,32,40,43^ sedation was measured using physiological factors monitored by technical approaches. The Neurosense^24,43^ assesses hypnotic depth of anesthesia by displaying two EEG signals and calculating several parameters, including the Wavelet Anesthetic Value for Central Nervous System (WAVcns), ranging from 100 (awake) to 0 (flat EEG).^24^

The Bispectral Index Score (BIS)^24,32,40^ is a non-invasive and validated instrument to measure the hypnotic effect of sedative and anesthetic medications, ranging from 100 (fully awake and aware) to 0 (brain death). Each patient is connected to a Quatro sensor applied to the forehead and analyses frontal EEG input using an algorithm.

Another reported non-invasive technique is the continuous monitoring of Heart Rate Variability (HRV) transformed into an Analgesia Nociception Index (ANI, 0–100),^24,43^ which assesses parasympathetic activity as a possible measure of nociception as HRV reflects the effect of the vagus nerve on the heart which is inhibited during pain. ANI has been shown to be effective in detecting pain in deeply sedated critically ill patients.^24^ It is based on the analysis of the respiratory fluctuations of heart rate that reflect the variability in the parasympathetic tone.^24,43^

#### Assessment responsibility and timing

Sedation was monitored by different professionals. Most assessments were performed by nurses^4,7,10,17,24,31,32,40,44^ with fewer assessments undertaken by physicians,^24,35,40^ researchers and palliative care professionals.^6^

Almost half of the articles included mentioned the timing of assessments. Daily assessment was common as a minimum requirement.^7,24,32,39,44^ Other articles reported hourly measurements,^13,42^ hourly during the first 4 h,^34^ 6 hourly until reaching adequate sedation,^16^ maximum of five times^31^ or six times per day.^32,40^ In the case of technical approaches for monitoring, continuous registration of values is described.^32,43^

#### Adverse events

During the administration, unintended effects of palliative sedation might occur. Only one study grades the severity of adverse events based on the Common Terminology Criteria for Adverse Events version 4.0.^13^ They considered the causality of adverse events when grading 3 or 4. They reported that apnea occurred in 1/32 patients receiving proportional sedation group and in 4/18 of the deep sedation group. No fatal events were considered as probably or definitely related to the intervention, and protocol-based sedation was continued in all cases. Other adverse events such as decreased respiratory rates (entire cohort of 32 patients^35^; (10/89 patients),^42^ decreased oxygen saturation (3/89 patients),^44^ and paradoxical agitation (3/89 patients)^44^ were reported. The majority of the studies did not explain how these adverse events were measured, only one referred to the use of oximeter for oxygen saturation.^44^ In the rest it can be deduced that adverse events were measured through observation as consisted on assessing and registering vital signs (i.e. respiratory rate).

One of the most controversial adverse events, raising ethical concerns, is whether palliative sedation hastens death. In this review, several articles demonstrated that palliative sedation does not shorten survival.^6,37,44^ Survival was calculated considering the period from hospitalization to death^6,44^ or from enrolment in the study to death.^37^ Azoulay et al.,^12^ analyzed the survival of palliative sedated patients considering who initiated the decision to use it (i.e. patient, medical staff or family) and the type of sedation, with no differences on survival. Finally, other study compared survival between the patients who were administered continuous deep sedation and those who were not.^41^ Survival was statistically significantly longer in the continuous deep sedation group than the non-continuous deep sedated group.^41^

## Discussion

The results of this review of 26 articles from 14 different countries, clearly demonstrates an increasing international interest in the use of palliative sedation. The majority of the articles report on studies conducted in a single site or several sites within the same country or region. Studies mainly included patients with cancer but some included patients with non-malignant conditions.

This review demonstrates improvements in comparison with the review of Brinkkemper^18^ as there is an increase in available and validated monitoring instruments of refractory symptoms and the effects of palliative sedation over the last ten years. Articles unanimously agree that refractory symptoms are a prerequisite when considering sedation. Articles tend to name the refractory symptoms, but often their assessment is not clearly reported with limited or no information on the evaluation instruments used or scores obtained. Among the instruments validated for palliative care and most frequently used are the Edmonton Symptom Assessment System (ESAS),51 the Memorial Delirium Assessment Scale (MDAS)55 and the Critical Care Pain Observational Tool (CPOT).48 The ESAS assesses a variety of symptoms, but it has been mainly used in the studies to assess pain while others used the CPOT; whereas delirium has been assessed with MDAS. These instruments are commonly used assessment instruments, suggesting that in clinical practice instruments are used to identify refractory symptoms. This practice is important as it allows comparison between studies and settings. In addition, when patients are unable to provide details about their symptoms (i.e. due to delirium, the depth of sedation), proxies can be an important source of information. Thus, it is also important that the symptom assessment instruments have been validated for proxy-as well as for self-assessment.

### Assessment of profound psychological or existential distress

It is noteworthy that occasionally profound psychological or existential distress are mentioned as reason for starting palliative sedation.^6,12,17^ No instruments to assess them have been identified in the review. This may be due to lack of awareness and underreporting related to professionals being more geared towards documenting physical sign and symptoms more than existential distress; rather than low incidence of this indication. There may be several explanations for this. There is a lack of consensus on a definition of existential suffering.^71^ Existential distress has been linked to aspects such as loss of personal meaning and purpose to life, fear of death, despair, hopelessness, loss of dignity, sense of isolation.^72,73^ No instruments were reported in the included articles. However, a review on available instruments to assess suffering for use in palliative care has identified instruments for assessing psycho-existential suffering.^71^ These instruments may be used to improve awareness in this area. Moreover, assessment of existential distress requires a complete multidimensional approach including psychologists, psychiatrists and/or spiritual caregivers, in order to identify it as a refractory situation.^71^

### Instruments to monitor level of sedation in palliative care context

Seven different instruments to monitor level of sedation have been identified in this review. Four of these are validated in palliative care, compared with the two, the Ramsay sedation scale, the RASS and the Communication Capacity Scale, reported in the 2013 published review.^18^ This provides more choice of instruments that can be utilized in palliative care. Two instruments assess the agitation level of the patient, which also assess level of sedation,^34^ and the Agitation Distress Scale.^39^ The RASS-PAL has received the highest rating on psychometric properties together with the Consciousness Scale for Palliative Care, according to a recent systematic review evaluating instruments to monitor level of consciousness on palliative patients.^73^ The other two instruments identified in the current review and validated for palliative care are the KNMG sedation score of the Royal Dutch Medical Association^62^ and the Consciousness Scale for Palliative care.^8^ The former being mainly used in Dutch contexts and the latter reported as easy to use.^66^ Articles identified did not discuss the use of neurological levels of somnolence, stupor and coma as a means for monitoring depth of sedation.

Technical approaches to assess physiological responses coming from anesthesiology are being used to assess level of sedation^6,32^ or parasympathetic activity.^24,43^ In a case report the use of Neurosense monitor was described by families as quite acceptable and non-intrusive.^43^ However, reliability of these methods has not been proven outside the controlled setting of an operating theatre.^32^ The technical equipment, but also the wide range of BIS values in deeply sedated and comfortable patients make its use in routine clinical practice unlikely.^74^

### Measuring palliative sedation effect

As the aim of palliative sedation is relief from refractory symptoms and not achievement of a specific level of consciousness,^3,75^ instruments that assess symptom relief or patient comfort are recommended. Two instruments for comfort assessment, the Discomfort Scale Dementia of Alzheimer Type (DS-DAT)^69^ and Patient Comfort Score^70^ were identified; the latter being validated in palliative care context. These instruments use observational criteria indicating that the patient seems relaxed, does not grimace and is not agitated.

### Adverse events

Potential adverse events need to be considered when assessing the effect of palliative sedation, but this review found little information relating to this and how these are assessed. The risk of hastening death is a serious adverse event, but a Cochrane review^5^ found no evidence that palliative sedation hastens death. Documentation of respiratory rates, blood pressure and cardiac arrest were reported as safety measures in one publication.^76^ Documenting vital signs has little consequences on these patients as there is a decline in them as part of the dying process and might lead even to wrong consequences, for example reduction or withdrawal of sedation in the dying patient when he needs it. This review has identified very few cases of adverse events being reported and even less information about how these are assessed. Future research should include systematic assessment and documentation of adverse events.

### Assessment of palliative sedation: expertise, timing, and proportionality

In the reviewed articles, assessment was conducted by healthcare professionals, mainly nurses. There is very limited information about professionals’ training to assess sedation and refractory symptoms. Only one article reports that regular in-service training and information sessions were provided to ensure competency and proficiency to undertake assessments.^32^ As many of the included studies were conducted within palliative care services, it might be assumed that the professionals were adequately trained for monitoring palliative sedation. However, there is an ongoing discussion in the literature about who should monitor sedation and about the need for consultation of a palliative care specialist for expert assessment of refractory symptoms.^3,77,78^ The use of validated instruments for palliative care patients is recommended, but a holistic clinical assessment needs to contain more than the use of them. Although, it is recommended that family members can provide input with the assessment of patients distress.^3^ Further studies are needed due to the limited evidence on this.^15^

There is broad consensus that patients should be assessed and monitored at the initiation as well as continuously throughout the sedation process. However, there was a wide range of assessment times reported in the literature. The EAPC framework recommended assessment initially at least once every 20 min until adequate sedation is achieved and subsequently at least three times per day.^3^ This is in line with a systematic review on published clinical guidelines that assessed recommendations on monitoring.^79^ Guidelines recommended frequent monitoring during initiation of palliative sedation with intervals of 15–30 min. For ongoing sedation, they reported monitoring intervals ranging from hourly to once a day.^79^ The clinical guidelines agreed that it is essential to monitor that the patient is comfortable, does not receive too much or too little sedation and that possible adverse events can be identified and acted on.^79,80^ It would be interesting to know if monitoring reported on papers is part of the daily clinical practice or as part of the study monitoring, as it may explain the variations.

The literature on sedation implicitly refers to continuous sedation: only a few articles referred to intermittent sedation.^8,9,31^ Even though, definitions of sedation deliberately made no distinction between continuous and intermittent, and light and deep sedation,^75^ there seems to be an underlying trend to assume that palliative sedation is always or mainly continuous deep sedation. The concept of proportionality is quite often mentioned in the literature, making a concept already implied in the definition more explicit,^4,8,12,75,81^ emphasizing this way that palliative sedation needs to be adjusted progressively to control the refractory symptom although it seems that often requires reaching deep sedation to manage the symptom.

The studies in this review investigated palliative sedation in an end-of-life context, though there was considerable variability on the timeframe. Sometimes palliative sedation was offered during a period where patients had an estimated prognosis of 6 months^12^ and in other studies 2 weeks (REF Abdul-Razzak era k 2019) or for the last hours of life.^30^ The tendency reported in the clinical cases is within days except in individual cases when intermittent sedation was administered for months (< 6 month).^9^ This has implications for the method and timing of assessment, as intermittent sedation may not need such a close assessment as continuous cases and can count with patient perspective. In consequence, the European Society for Medical Oncology (ESMO) guideline suggests that patient monitoring should be determined by the clinical situation,^82^ taking into account also the level of unconsciousness.

### What this study adds

This study updates the information about instruments available to assess the effect of palliative sedation, beyond observational scales to measure the level of sedation. It provides information on assessment tools used to measure other potential effects of palliative sedation such as symptom control, comfort or related-adverse events, specifying if they are validated for palliative care context. It adds a comprehensive view of the assessment, considering the expertise and involvement of people on it and the timing. The EAPC framework on palliative sedation highlighted the need to define the quality of sedation.^3^ For this aim, a well oriented and comprehensive assessment is needed. Assessing refractory symptom relief, patient comfort with the minimum decrease on consciousness lowering of conscious level and adverse events, can be a way to describe the quality of the intervention in clinical practice. This study provides information about it and suggestions to improve palliative sedation assessment. However, this study do not want to be prescriptive considering that instruments need to be clinically applied being available in local language and being culturally adequate. In order to specify further recommendations, it would be interesting to consider if the tools are available in the language needed and this was out of the scope of the review. Further studies should be done measuring the effects of palliative sedation with more adequate assessment strategies to show more clearly the contribution of it to patient comfort and symptom relief.

### Strengths and limitations

Rigorous methodological steps were used to decrease the risk of bias, for example using independent and blinded assessment of articles. However, a few limitations apply to this review. Only articles in English were included and there may be relevant articles published in other languages (e.g. French) that have not been included. However, this review provides information from 14 countries. The different research methods used in the articles, and variability in the assessments reported, complicated compilation of findings and did not allow for meta-analysis. The under representation of vulnerable groups and cultural minorities in the studies is also a limitation. One article mentioned that they did not include patients of indigenous descent as it required additional approval and based on the very small number of indigenous people usually admitted to the unit.^32^

## Conclusion

The review identified validated instruments in palliative care context that allow assessing the effect of palliative sedation including its outcomes, relief from refractory symptoms and patient’s comfort. These instruments should be used within an expert interdisciplinary team who can provide a complete clinical assessment. A standardized approach for assessment, including timing and documentation, and adequate training for healthcare professionals is needed to improve both clinical practice and support comparison between research studies. Adverse events are not commonly reported on palliative sedation, possible risk of hastening death is the most studied. Future studies need to specify the systematic assessments conducted including possible adverse events.

## Supplemental Material

sj-docx-1-pmj-10.1177_0269216320974264 – Supplemental material for How to measure the effects and potential adverse events of palliative sedation? An integrative review Click here for additional data file. Supplemental material, sj-docx-1-pmj-10.1177_0269216320974264 for How to measure the effects and potential adverse events of palliative sedation? An integrative review by Alazne Belar, María Arantzamendi, Sheila Payne, Nancy Preston, Maaike Lee-Rijpstra, Jeroen Hasselaar, Lukas Radbruch, Michael Vanderelst, Julie Ling and Carlos Centeno in Palliative Medicine
