# Supplementary material for: How to measure the effects and potential adverse events of palliative sedation? An integrative review
Source: Palliat Med. 2020 Dec 14;35(2):295–314. doi: 10.1177/0269216320974264 (PMC7897792; doi:10.1177/0269216320974264)
Supplement: sj-docx-1-pmj-10.1177_0269216320974264 – Supplemental material for How to measure the effects and potential adverse events of palliative sedation? An integrative review [file sj-docx-1-pmj-10.1177_0269216320974264.docx]

SUPPLEMENTARY FILES

**Appendix 1. Strategy string**

**Pubmed**

- (sedation[All Fields] AND ("Palliative Care"[Mesh] OR "Terminal Care"[Mesh])) AND ("Assessment"[Journal] OR "assessment"[All Fields]) AND (("2010/01/01"[PDAT]: "2020/05/28"[PDAT]) AND English[lang] AND "adult"[MeSH Terms])

**Cochrane Library**

## Sedation in Record Title AND "palliative care" in Title Abstract Keyword OR "terminal care" in Title Abstract Keyword AND assessment in Title Abstract Keyword. Limits: 2010/01/01-2020/05/28

**Cinahl**

- TI sedation AND AB palliative care OR AB terminal care AND AB assessment. Date: 20100101-20200528; Age: adults; Language: English

**Medline (WoS)**

- (Sedation) AND TOPIC: ("palliative care") OR TOPIC: ("terminal care") AND TITLE: (assessment). PUBLICATION YEARS: (2020 OR 2019 OR 2018 OR 2017 OR 2016 OR 2015 )

**Appendix 2. Predefined data extraction sheet for integrative review**

| **Author, year** |
| --- |
| **Objectives** *(in case it has)* |
| **Study design** |
| **Setting & or context** |
| **General patients characteristics** (ie: specify gender, referring to place of residence,  race/ethnicity/culture/language, occupation, gender/sex, religion, education) |
| **Theoretical models** used (if any) |
| **Definition of Sedation** (Author) and wording used. |
| **Refractory symptoms** measured: comfort, symptom,… |
| Validated instruments used to measure refractory symptoms |
| Domains included |
| Are they validated for PC? |
| Are they validated in general care? |
| What is the most important to know?  Background of users (ie: nurse, physician…)  Feasibility to use in different settings  Who is expected to answer the instrument |
| Non validated instruments |
| **Palliative sedation** |
| Palliative Sedation Validated instruments used |
| Domains included |
| Are they validated for PC? |
| Are they validated in general care? |
| What is the most important to know?  Background of users (ie: nurse, physician…)  Feasibility to use in different settings  Who is expected to answer the instrument |
| Registration of vital signs (eg: respiratory rate, blood pressure, heart rate, temperature… for safety reasons or as indications of patient distress |
| Non validated instruments |
| **Subjective perceptions** |
| Experiences |
| Satisfaction |
| Perception |
| **Deviant data (if any)** |
| **Complications of palliative sedation** |
| **Comments of the reviewer** |
| **Methodological strengths and limitations** |
